# Residential Radon Levels and Ovarian Cancer Among Postmenopausal Women

**DOI:** 10.1001/jamanetworkopen.2026.8641

**Published:** 2026-04-10

**Authors:** Mark R. Williamson, Eric A. Whitsel, Richard L. Smith, Jason M. Collins, James D. Stewart, Su Yon Jung, Holly R. Harris, Marina Oktapodas Feiler, JoAnn E. Manson, Lihong Qi, Gary G. Schwartz

**Affiliations:** 1 Department of Population Health, School of Medicine & Health Sciences, University of North Dakota, Grand Forks; 2 Department of Epidemiology, Gillings School of Global Public Health, University of North Carolina, Chapel Hill; 3 Department of Medicine, School of Medicine, University of North Carolina, Chapel Hill; 4 Department of Statistics & Operations Research, Gillings School of Global Public Health, University of North Carolina, Chapel Hill; 5 Department of Biostatistics, Gillings School of Global Public Health, University of North Carolina, Chapel Hill; 6 Department of Epidemiology, Fielding School of Public Health, Jonnson Comprehensive Cancer Center, University of California, Los Angeles; 7 Program in Epidemiology, Public Health Sciences Division, Fred Hutchinson Cancer Center, Seattle, Washington; 8 Department of Epidemiology and Environmental Health, School of Public Health and Health Professions, University at Buffalo, Buffalo, New York; 9 Department of Medicine, Brigham and Women’s Hospital, Harvard Medical School, Harvard University, Boston, Massachusetts; 10 Department of Public Health, School of Medicine, University of California, Davis

## Abstract

**Importance:**

Few environmental risk factors for ovarian cancer have been discovered. Women exposed to ionizing radiation from the atomic bomb during World War II experienced an increased risk of ovarian cancer. Today, the largest source of ionizing radiation is radon gas in the home, but whether ionizing radiation is associated with increased risk of ovarian cancer more broadly is unknown.

**Objective:**

To evaluate whether higher home radon levels are associated with increased risk of ovarian cancer.

**Design, Setting, and Participants:**

This prospective cohort study included 127 547 women from the Women’s Health Initiative, including 40 clinical centers across the US, with outcomes followed up for 31 years (1993-2024). Postmenopausal women aged 50 to 79 years were enrolled in an observational study or 1 or more randomized clinical trials. All cases of ovarian cancer were physician adjudicated.

**Exposure:**

Radon measurements from the 1993 US Geological Survey, classified into low (<2 pCi/L), medium (2-4 pCi/L), and high zones (>4 pCi/L), were linked with the geocoded home addresses of participants at baseline (1993-1998).

**Main Outcomes and Measures:**

Hazard ratios (HRs) for ovarian cancer, adjusted for covariates with 95% CIs.

**Results:**

Among the 127 547 women (mean [SD] age, 63.1 [7.2] years) with available radon zone values, 1645 incident ovarian cancers and 1048 ovarian cancer deaths were observed over a mean (SD) follow-up of 17.7 (8.4) years. After adjustment for covariates, the HR for all ovarian cancers for women living in the medium radon zone compared with women living in the low radon zone was not significantly higher (HR, 1.13 [95% CI, 1.00-1.29]). However, the HR was significantly higher for women living in the high radon zone compared with those living in the low radon zone (HR, 1.31 [95% CI, 1.11-1.54]). Similar findings were observed for the most common histologic type, serous ovarian cancer, for which the HR in the medium zone was 1.06 (95% CI, 0.88-1.27) and the HR in the high zone was 1.38 (95% CI, 1.09-1.74). Ovarian cancer mortality also was significantly higher in the high radon zone compared with the low radon zone (HR, 1.31 [95% CI, 1.07-1.60]). Sensitivity analyses using 3 alternate radon measures produced similar results.

**Conclusions and Relevance:**

In this large, prospective cohort of postmenopausal women, the risks of ovarian cancer incidence and mortality were significantly higher for women living in homes in the high radon zone. Residential radon is a ubiquitous and modifiable risk factor. This is the first epidemiologic study of radon and ovarian cancer among postmenopausal women to date, and its findings suggest a potential target for mitigating cancer risk.

## Key Points

**Question:**

Are residential radon levels associated with an increased risk of ovarian cancer?

**Findings:**

This cohort study of 127 547 women with available radon zone values from the Women’s Health Initiative found that those who lived in homes with higher radon levels had a significantly increased risk of ovarian cancer.

**Meaning:**

This study suggests that radon, a preventable and common exposure, may be associated with an increased risk of ovarian cancer.

## Teaser

This cohort study uses data from the Women’s Health Initiative to evaluate whether higher residential radon levels are associated with an increased risk of ovarian cancer among postmenopausal women.

## Introduction

Ovarian cancer is the fifth leading cause of cancer deaths among US women, with an estimated 20 890 new cases and 12 730 deaths in 2025.^1^ The etiology of ovarian cancer is incompletely understood. Approximately 25% of cases have a strong genetic component, predominantly linked with variants in the *BRCA1/2* (OMIM 113705/600185) genes. Variables that are associated with a greater lifetime number of ovarian cycles (eg, late age at menopause) are associated with increased risk of ovarian cancer. Similarly, factors associated with fewer ovarian cycles (eg, oral contraceptive use) are associated with reduced risk.^2^ There are several histologic types of ovarian cancer, with different cells of origin and distinct etiologies.^3^ However, approximately 90% of ovarian cancers are epithelial malignant neoplasms, and 70% to 80% of these are high-grade serous carcinomas, the most lethal histologic type.^4^ Because the symptoms of early-stage ovarian cancers are nonspecific, and because there are no effective screening modalities, most ovarian cancers are detected at advanced stages, for which survival is poor.

Few environmental causes of ovarian cancer are known.^3^ The use of estrogen without progesterone (menopausal hormone therapy) increases risk.^5^ World War II Japanese atomic bomb survivors experienced an increased risk of ovarian cancer, but whether ionizing radiation increases the risk of ovarian cancer among women not exposed to the atomic bomb has not been well studied.^6,7^ The largest source of ionizing radiation for most individuals is from radon, a naturally occurring gas produced by the decay of uranium and other radioactive elements present in soil.^8^ The gas enters homes through cracks in the foundation and can accumulate indoors. Radon gas is soluble in blood and can reach into internal organs.^9,10^ However, the main radiation dose from radon comes from its decay products, radon progeny, which are solids that attach to dust in air.^9^ Radon progeny emit high-energy α particles that can cause cancer when inhaled. Radon is an established cause of lung cancer and a suspected cause of other malignant neoplasms.^11^

The Women’s Health Initiative (WHI) is a large, longitudinal cohort of postmenopausal women with extensive data on health and residential history.^12^ We used WHI data to examine the association of ovarian cancer with residential radon levels, including overall incidence, overall mortality, and incidence stratified by histologic type.

## Methods

### Study Population

Between 1993 and 1998, 40 WHI clinical centers across the US enrolled postmenopausal women aged 50 to 79 years into an observational study (n = 93 676) or 1 or more overlapping, randomized clinical trials (n = 68 132) of menopausal hormone therapy, calcium and vitamin D supplementation, and diet modification. The total cohort of 161 808 women has been followed up for 31 years, through 2024. The WHI was approved by the Fred Hutchinson Cancer Center institutional review board in accordance with US Department of Health and Human Services regulations at 45 CFR 46. All participants provided written informed consent to participate and for review of their medical records. The Fred Hutchinson Cancer Research Center has an approved Federal-wide Assurance on file with the Office for Human Research Protections under assurance number 0001920. This study followed the Strengthening the Reporting of Observational Studies in Epidemiology (STROBE) reporting guideline for cohort studies.

### Exclusions

We excluded 2490 women from the analytic sample with self-reported or missing history of ovarian cancer at enrollment or randomization (baseline), 690 with missing follow-up, and 31 077 women who underwent a bilateral oophorectomy or were missing history of oophorectomy, yielding an eligible population of 127 551 women (78.8% of the total) followed up through July 12, 2024. Of those, radon zone values were available for 127 547 (99.9%).

### Exposure: Radon

Our radon measurements follow methods previously reported.^13^ In brief, we used measurements from the 1993 US Geological Survey (USGS) and linked them with the geocoded home addresses of participants at baseline (1993-1998).^14^ We used the USGS radon zones,^15^ which are 3 categories based on a unitless USGS radon index (range, 5-15), which itself is based on several factors including short-term radon measurements, house characteristics, geology, soil permeability, and aerial radioactivity.^16,17^ We refer to the 3 zones, in picocuries per liter of air, as low (<2 pCi/L), medium (2-4 pCi/L), and high (>4 pCi/L) (1 pCi/L = 37 Bq/m^3^). The highest value (ie, >4 pCi/L) represents the US Environmental Protection Agency (EPA) action level under its National Radon Action Plan.^18,19^

### Outcome: Ovarian Cancer

Study physicians reviewed, classified, and adjudicated incident ovarian cancer diagnoses or deaths from ovarian cancer for clinical trial and observational study participants.^20^ Most high-grade serous ovarian cancers are thought to originate in the fallopian tubes and grow on the ovary and/or peritoneum.^21^ We included fallopian tube cancers (labeled as genital organ cancers in the WHI) and peritoneum cancers with ovarian cancers under the following *International Classification of Diseases for Oncology, Third Edition* (*ICD-O-2*) codes: 56.9, 48, 48.1, 48.2, 48.8, 57, 57.1, 57.3, 57.4, 57.8, and 57.9. The WHI variable MRPHHISTBDESC (morphology-histology/behavior description) designated histologic subtypes. All fallopian tube and peritoneum cancers were treated as serous. Nonserous cancers included cases with other descriptions and cases without an *ICD-O-2* code or histologic description. All cases of ovarian cancer were adjudicated centrally.

### Covariates

Variables within the WHI dataset include trial design characteristics, sociodemographic data, and behavioral, clinical, and reproductive data. Design characteristics describe the clinical trial participation (vs observational trial only) and hormone therapy trial arm assignment (hysterectomy with estrogen treatment, hysterectomy with placebo, intact uterus with estrogen and progestin treatment, intact uterus with placebo, or not randomized). Women who enrolled in the WHI study, in and outside the trials, may have used estrogens from other clinical sources. This estrogen use was documented at baseline.

Sociodemographic characteristics included age, race (White or race other than White [American Indian or Alaska Native, Asian, Black, Native Hawaiian or Other Pacific Islander, >1 race, or unknown or not reported]), ethnicity (Hispanic or non-Hispanic), US census region (northeast, south, midwest, or west), education (<college or ≥college), occupation (homemaker or other), and neighborhood socioeconomic status (summary *z* score).^22^ Race and ethnicity were self-reported using preset, structured response options. Given the small cell counts across some categories, race and ethnicity were dichotomized. Data on race and ethnicity were collected, in part, to fulfill federal mandates on inclusion of members of racial and ethnic minority groups and to identify health disparities among diverse populations. Behavioral, clinical, and reproductive data included smoking status (ever or never), body mass index (calculated as weight in kilograms divided by height in meters squared), oral contraceptive use (ever or never), age at menarche (≤12 vs ≥13 years), parity (the number of full-term pregnancies; none, 1-2, or ≥3), age at menopause (years), breastfeeding months (none, 1-6, or ≥7), partial oophorectomy status (none or 1 or unsure), and hysterectomy status (yes or no). Many women who reported a history of hysterectomy still retained their ovaries.

### Statistical Analysis

We calculated follow-up time in years as the interval between the date of enrollment or randomization and the date of first occurrence of incident ovarian cancer, loss to follow-up, death, or end of follow-up (July 2024). We estimated the ovarian cancer (cumulative) incidence proportion and rate by USGS radon zone as the unadjusted number of incident ovarian cancers per 1000 women at risk and per 100 000 woman-years, respectively. After confirming the proportional hazards assumption by comparing the Schoenfeld residuals against the transformed time (χ^2^ = 0.27; *P* = .87), we estimated the relative hazard of ovarian cancer in the medium and high radon zones relative to the low zone as a hazard ratio (HR) and 95% CI using Cox proportional hazards regression, adjusting for covariates. We estimated HRs for ovarian cancer separately for ever smokers and never smokers.

In sensitivity analyses, we examined the sensitivity of the fully adjusted estimates to substituting the USGS radon index; EPA radon zones (an extrapolation of the USGS radon zones to county boundaries)^14^; and the E. O. Lawrence Berkeley National Laboratory (LBL) county-level geometric mean for the USGS radon zones (bayesian hierarchical modeling of mean radon concentrations across all living areas for 1 year).^23^ Additional analyses included examining sensitivity to clinical center–level random effects; multiple imputation of missing data using the MICE (multivariate imputation by chain equations) function in R, version 4.4.1 (R Project for Statistical Computing) (eTable 1 in Supplement 1); stratifying by serous vs nonserous tumors, using a joint Cox proportional hazards regression model that accommodates disease subtypes^24^; examining mortality; and stratifying by family history of breast cancer. All *P* values were from 2-sided tests and results were deemed statistically significant at *P* < .05.

## Results

### Participant Characteristics

Radon zone values were available for 127 547 women (mean [SD] age, 63.1 [7.2] years; 5771 Hispanic [4.5%], 109 122 White [85.6%], and 18 429 race other than White [14.4%]) from the WHI (Table 1). At baseline, 22.1% of participants (28 225) lived in high radon zones, 37.7% (48 145) lived in medium radon zones, and 40.1% (51 177) lived in low radon zones (Figure; Table 2). A total of 54 413 women (42.7%) were randomized to 1 or more of the WHI clinical trials, and 3223 (2.5%) to 8359 (6.6%) were randomized to each of the 4 hormone therapy trial arms (Table 1). Most participants were college educated (99 556 [78.1%]) and employed outside the home (116 576 [91.4%]). Ever smoking was common (61 977 [49.2%]), and the mean (SD) body mass index was 27.9 (5.9). A total of 53 813 women (42.2%) reported contraceptive use. Most (69 959 [55.1%]) had 3 or more full-term pregnancies, experienced menarche at 13 years of age or older (67 076 [52.6%]), and breastfed for at least some time (65 575 [51.4%]). The mean (SD) age at menopause was 49.2 (5.8) years. Partial oophorectomy was reported by 15 628 included participants (12.3%), and hysterectomy by 36 446 (28.6%). Participants living at the highest radon zone (>4 pCi/L) were more likely to be White, non-Hispanic, residents of the northeast or midwest, and socioeconomically advantaged (eTable 2 in Supplement 1). Of 1657 tumors, 785 (47.4%) were serous, including tumors designated as generic, unknown, or other less common subtypes (eTable 3 in Supplement 1). Considering only tumors with identified histotype (serous, endometrioid, mucinous, and clear cell), we found that 78.7% (785 of 998) were serous.

**Table 1.** Characteristics of Participants at the Baseline Visit (Women’s Health Initiative, 1993-1998)

Characteristic	No. (%) (N = 127 547)
Study design	
Clinical trial participation (yes)^a^	54 413 (42.7)
Hormone replacement trial arm	
Not randomized	104 723 (82.1)
Hysterectomy: estrogen treatment	3272 (2.6)
Hysterectomy: placebo	3223 (2.5)
Uterus: estrogen plus progestin treatment	8359 (6.6)
Uterus: placebo	7974 (6.3)
Sociodemographic characteristics	
Age, mean (SD), y	63.1 (7.2)
Race^b^	
White	109 122 (85.6)
Race other than White^c^	18 429 (14.4)
Ethnicity^b^	
Hispanic	5771 (4.5)
Non-Hispanic	121 780 (95.5)
US Census region	
Northeast	30 203 (23.7)
South	32 166 (25.2)
Midwest	28 501 (22.3)
West	36 681 (28.8)
Education (<college)	27 991 (22.1)
Occupation (homemaker)	10 971 (9.2)
Neighborhood SES, mean (SD), *z* score	0.1 (5.4)
Behavioral, clinical, and reproductive characteristics	
Ever smoker^d^	61 977 (49.2)
BMI, mean (SD)	27.9 (5.9)
Contraceptive use (ever)	53 813 (42.2)
Parity	
None	14 605 (11.5)
1-2	42 423 (33.4)
≥3	69 959 (55.1)
Menarche (age ≤12 y)	60 471 (47.5)
Age at menopause, mean (SD), y	49.2 (5.8)
Breastfeeding mo	
None	60 537 (48.0)
1-6	32 580 (25.8)
≥7	32 995 (26.2)
Partial oophorectomy (1 or unsure)	15 628 (12.3)
Hysterectomy (yes)	36 446 (28.6)

Abbreviations: BMI, body mass index (calculated as weight in kilograms divided by height in meters squared); SES, socioeconomic status.

^a^ Multiple clinical trials were done in the Women’s Health Initiative, beyond the hormone replacement trial.

^b^ Given the small cell counts across some categories, race and ethnicity were dichotomized.

^c^ Race other than White included American Indian or Alaska Native, Asian, Black, Native Hawaiian or Other Pacific Islander, more than 1 race, and unknown or not reported.

^d^ Includes 8968 (7.1%) current and 53 009 (42.0%) former smokers.

**Figure.**
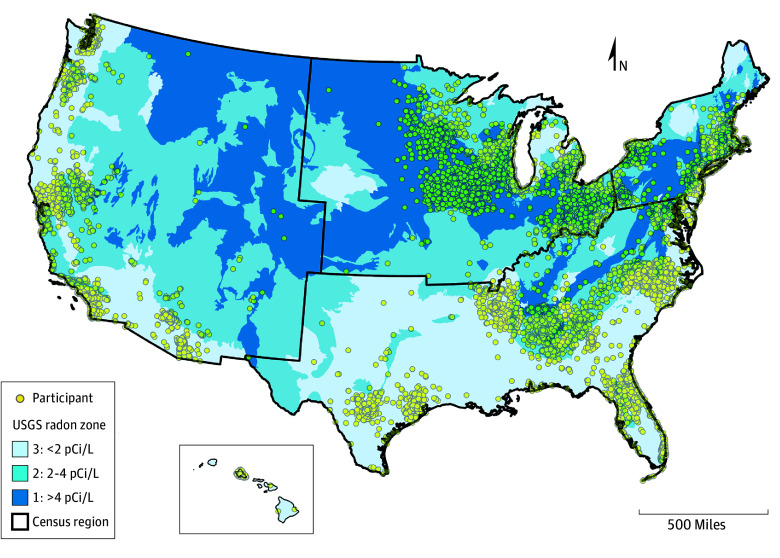
Map Showing Women’s Health Initiative Participant Distribution and Radon Hazard Distribution of participants at the baseline visit, by US Geological Survey (USGS) radon zone and US Census region (Women’s Health Initiative, 1993-1998). The map includes 127 543 of the 127 547 participants (yellow dots) used in the analysis (4 participants have no location code).

**Table 2.** Absolute Risk of Ovarian Cancer by USGS Radon Zone (Women’s Health Initiative, 1993-2024)

Radon zone (level in pCi/L)	Women at risk, No. (%)	Follow-up, mean (SD), y	Ovarian cancer, No. (%)	Incidence	
				Proportion^a^	Rate^b^
High (>4)	28 225 (22.1)	18.3 (8.1)	408 (24.8)	14.5	76.6
Medium (2-4)	48 145 (37.7)	17.7 (8.3)	627 (38.1)	13.0	73.2
Low (<2)	51 177 (40.1)	17.4 (8.5)	610 (37.1)	11.9	70.1
All (any)	127 547 (100)	17.7 (8.4)	1645 (100)	12.9	72.8

Abbreviation: USGS, US Geological Survey.

^a^ Per 1000 women at risk.

^b^ Per 100 000 woman-years.

### Covariates

We estimated HRs for ovarian cancer in association with known risk factors to adjust for them as potential confounders. HRs for ovarian cancer increased with age, decreased with increasing number of full-term pregnancies, decreased with partial oophorectomy and hysterectomy, and differed across region and hormone therapy trial arm. These covariate results replicate findings from previous studies from the WHI^5^ (eTable 4 in Supplement 1).

### Ovarian Cancer Incidence Proportion and Rate

A total of 1645 incident ovarian cancers and 1048 ovarian cancer deaths were observed over a mean (SD) follow-up of 17.7 (8.4) years (Table 2). The unadjusted incidence proportion was 12.9 ovarian cancers per 1000 women at risk and, the rate was 72.8 incident ovarian cancers per 100 000 woman-years. Both measures were higher in the high radon zone than in the medium and low radon zones, with 14.5 incident ovarian cancers per 1000 women at risk in the high zone, 13.0 incident ovarian cancers per 1000 women at risk in the medium zone, and 11.9 incident ovarian cancers per 1000 women at risk in the low zone and with 76.6 incident ovarian cancers per 100 000 woman-years in the high zone, 73.2 incident ovarian cancers per 100 000 woman-years in the medium zone, and 70.1 incident ovarian cancers per 100 000 woman-years in the low zone.

### Relative Hazard of Ovarian Cancer

Before adjustment, the relative hazards of ovarian cancer were higher in the medium vs the low zone (HR, 1.04 [95% CI, 0.93-1.16]) and in the high vs the low zone (HR, 1.09 [95% CI, 0.96-1.23]) (Table 3). After cumulative adjustment, the HR in the medium zone was not significantly higher than in the low zone (HR, 1.13[95% CI, 1.00-1.29]). Conversely, the HR in the high zone was significantly higher than in the low zone (HR, 1.31 [95% CI, 1.11-1.54]).

**Table 3.** Relative Risk of Ovarian Cancer by USGS Radon Zone (Women’s Health Initiative, 1993-2024)

Cumulative adjustment	Radon zone, hazard ratio (95% CI)			
	Low (<2 pCi/L)	Medium (2-4 pCi/L)	High (>4 pCi/L)
None	1.00 [Reference]	1.04 (0.93-1.16)	1.09 (0.96-1.23)
Plus study design^a^	1.00 [Reference]	1.04 (0.93-1.17)	1.09 (0.96-1.24)
Plus sociodemographic characteristics^a^	1.00 [Reference]	1.13 (1.00-1.27)	1.25 (1.07-1.46)
Plus clinical, behavioral, and reproductive characteristics^a^	1.00 [Reference]	1.13 (1.00-1.29)	1.31 (1.11-1.54)

Abbreviation: USGS, US Geological Survey.

^a^ See Table 1 for variables included.

### Sensitivity Analyses

The pattern in the HRs remained when including clinical center–level random effects and multiply imputing missing values. For alternate EPA radon measures, the EPA’s high radon zone (zone 1) had a higher HR than the low zone (zone 3), while the medium zone (zone 2) had the same HR as the low zone (Table 4). For the noncategorical radon measures, the hazards increased 3% per unit increase in the USGS radon index and 11% per pCi/L increase in the LBL geometric mean.

**Table 4.** Sensitivity Analyses of Association Between Radon Zone and Ovarian Cancer Risk, Fully Adjusted Model (Women’s Health Initiative, 1993-2024)

Characteristic	Radon zone, hazard ratio (95% CI)		
	Low (<2 pCi/L)	Medium (2-4 pCi/L)	High (>4 pCi/L)
Main analysis	1.00 [Reference]	1.13 (1.00-1.29)	1.31 (1.11-1.54)
**Sensitivity analyses**			
Adjustment for clinical center	1.00 [Reference]	1.07 (0.93-1.22)	1.16 (0.99-1.36)
Adjustment for multiple imputation	1.00 [Reference]	1.09 (0.97-1.23)	1.19 (1.02-1.38)
Radon exposure substitution			
EPA radon zone	1.00 [Reference]	1.00 (0.88-1.13)	1.16 (0.99-1.35)
USGS radon index	NA	1.03 (1.00-1.06)	NA
LBL geometric mean	NA	1.11 (0.99-1.25)	NA
**Other analyses**			
Serous only	1.00 [Reference]	1.06 (0.88-1.27)	1.38 (1.09-1.74)
Joint Cox proportional hazards regression model of histologic characteristics^a^			
Serous	1.00 [Reference]	1.10 (0.92-1.32)	1.48 (1.20-1.82)
Nonserous	1.00 [Reference]	1.05 (0.83-1.33)	0.78 (0.60-1.33)
Mortality only	1.00 [Reference]	1.11 (0.95-1.30)	1.31 (1.07-1.60)
No breast cancer family history only	1.00 [Reference]	1.11 (0.88-1.41)	1.28 (0.95-1.74)
Positive breast cancer family history only	1.00 [Reference]	1.10 (0.81-1.47)	1.63 (1.13-2.34)

Abbreviations: EPA, US Environmental Protection Agency; LBL, E.O. Lawrence Berkeley National Laboratory; NA, not applicable; USGS, US Geological Survey.

^a^ Significant likelihood ratio between serous and nonserous strata (likelihood ratio test, 155.1; *P* < .001).

Restricting ovarian cancer cases to serous cancers (n = 676) and using the fully adjusted model, we found that the HRs were 1.06 (95% CI, 0.88-1.27) in the medium zone and 1.38 (95% CI, 1.09-1.74) in the high zone (Table 4). In a joint Cox proportional hazards regression model of serous and nonserous tumors, the HR for serous tumors was not significantly higher in the medium zone (1.10 [95% CI, 0.92-1.32]) but was significantly higher in the high zone (1.48 [95% CI, 1.20-1.82]). Conversely, the HRs for nonserous tumors (n = 761), including nonserous and unclassified, did not differ by zone (medium zone: HR, 1.05 [95% CI, 0.83-1.33]; high zone: HR, 0.78 [95% CI, 0.60-1.33]). The joint Cox proportional hazards regression model had a significant likelihood ratio between serous and nonserous strata (likelihood ratio test, 155.1; *P* < .001). When we substituted ovarian cancer mortality for incidence, the HRs in the medium and high zones were nearly identical to those for the incidence data (medium zone: HR, 1.11 [95% CI, 0.95-1.30]; high zone: HR, 1.31 [95% CI, 1.07-1.60]); the HR was statistically significant for the high zone. Stratification by smoking status showed similar HRs for both groups: for ever smokers in the medium zone, the HR was 1.12 (95% CI, 0.94-1.33), and for ever smokers in the high zone, the HR was 1.28 (95% CI, 1.03-1.60), whereas for never smokers in the medium zone, the HR was 1.16 (95% CI, 0.97-1.39), and for never smokers in the high zone, the HR was 1.35 (95% CI, 1.06-1.72).

Approximately half of the participants had missing data for family history of breast cancer. In a model restricted to women with no family history (n = 406), the HRs in the medium and high zones were elevated nonsignificantly (medium zone: HR, 1.11 [95% CI, 0.88-1.41]; high zone: HR, 1.28 [95% CI, 0.95-1.74]) (Table 4). Conversely, in a model restricted to women with a positive family history of breast cancer (n = 272), the medium zone had a nonsignificantly elevated HR (1.10 [95% CI, 0.81-1.47]), and the high zone had a significantly elevated HR (1.63 [95% CI, 1.13-2.34]).

## Discussion

The goal of this study was to examine ovarian cancer incidence and mortality in the WHI cohort in association with the level of radon at home. The mean age of WHI participants at baseline, 63 years, is the mean age at ovarian cancer diagnosis, making this cohort well suited to test hypotheses about ovarian cancer.^4^ Our most important findings are that, adjusted for covariates, the risk of ovarian cancer increased significantly with increasing radon exposure. Compared with the low radon zone, for all ovarian cancers, the HRs were higher in the high zone. We found similar results for serous tumors analyzed separately. Mortality rates for ovarian cancer also were significantly higher in the high radon zone. To our knowledge, this is the first epidemiologic study of radon and ovarian cancer at the individual level.

A previous ecologic study that used data from US states reported a negative correlation between radon and ovarian cancer incidence rates.^25^ However, that report is limited by small sample size, the use of group-level data, and the inability to control confounding.

The association between radon level and ovarian cancer conceivably could be confounded by smoking, which was measured as a binary variable and does not capture smoking intensity. However, stratification by smoking status showed similar HRs for both groups. Smoking prevalence has historically been highest in Appalachia and southern states. Our findings are unlikely to be influenced by regional differences in smoking as our models included smoking and region as covariates. More importantly, smoking does not appear to be a risk factor for serous ovarian cancers.^26,27^

Participants in the highest radon zone were more likely to be White and socioeconomically advantaged, raising the possibility that the association between radon and ovarian cancer incidence could reflect greater access to care. Although disease stage at diagnosis may be earlier among economically advantaged women, the incidence of ovarian cancer does not appear to be greater among women with higher socioeconomic status.^28^ Our finding of significantly increased ovarian cancer mortality in the high radon zone also is inconsistent with greater access to care.

A joint Cox proportional hazards regression model of serous and nonserous tumors showed an elevated HR for serous tumors in the high zone; the HR for nonserous tumors was not elevated. These findings are consistent with our current understanding that serous and nonserous ovarian cancers have different etiologies.^3^ Alternative statistical models using the USGS radon index (the underlying source of the USGS radon zones) and the LBL geometric data similarly found that the HR increased with radon index values and mean values.

Because a family history of breast cancer, a proxy for *BRCA* variants, is associated with increased ovarian cancer risk, we examined models for positive and negative family history groups separately. The HR for the positive family history group for the high zone, 1.63 (95% CI, 1.13-2.34), was the highest of any of our models. This high HR could reflect the nature of *BRCA* variants, which confer a decreased ability to repair DNA damage, including damage caused by ionizing radiation.^29,30^

The etiology of ovarian cancer is poorly understood. Prominent hypotheses involve the initiation of variants in ovarian cells (eg, via repeated ovulation) and the promotion of proliferation of mutated cells via hormones.^31,32,33^ Radon can exert both genotoxic and hormonal effects. *Radon* is a common shorthand term for radon gas and its decay products (progeny); the latter are solids that attach to fine particulates in air.^9^ In laboratory studies, radon gas at low concentrations is mutagenic in ovarian-derived cells.^34,35^ Particulate air pollution is reported to increase the risk of ovarian cancer, and direct evidence of air pollution (black carbon particles) has been found in human ovaries and follicular fluid.^36,37,38^ Particulate air pollution could act as a “Trojan horse” to convey radon progeny to ovarian tissues. Long-term radon exposure also might influence ovarian cancer hormonally. Halawa^39^ studied 26 postmenopausal women at a radon spa who inhaled radon repeatedly at nanocurie per liter levels (ie, 1000 times the levels in most US homes). Radon spas are facilities where participants are exposed to radon gas via balneotherapy (bathing in radon-rich water) and inhaling radon-infused air. Radon exposure is believed to provide relief from arthritis, rheumatic diseases, and other chronic conditions.^40^ After 18 one-hour inhalation treatments, serum estradiol increased 70% over pretreatment levels, estriol increased 90% over pretreatment levels, serum luteinizing hormone levels increased by 40%, and follicle-stimulating hormone levels increased by 80%. No changes were observed among 8 untreated, postmenopausal women.

### Strengths and Limitations

Our study has several strengths. First, the WHI is a well-characterized longitudinal study, and all ovarian cancer cases were centrally adjudicated. Second, our radon metrics are well established and have been used successfully in other studies, including those of WHI participants. Third, sensitivity analyses showed the same trend of increasing HRs with increasing radon zone that we observed for ovarian cancer incidence and mortality, indicating that our findings are not dependent on a particular model of exposure estimation.

Our study also has several limitations. First, our radon measurements were estimates based on physical factors. We had no information on whether homes had been remediated for radon. The number of remediated homes is small, as only 18% of US homes have ever been tested for radon.^41^ Second, the geocoded locations of WHI participants at baseline may not reflect participants’ predominant radon exposure. Although participants’ change in residence may have introduced error, the WHI cohort has been residentially stable since inception; typical participant moves have been over very short distances and between socioeconomically similar neighborhoods, factors that likely are associated with participant retention–enhancing criteria at the time of recruitment. Residential radon levels also are relatively stable over time; the reported year-to-year variability of repeated measurements in individual homes was 17%.^42^ Our models were sensitive to the clinical center, suggesting that unknown environmental factors may exist. Third, because histologic data were missing for some cases, our ability to examine histotype-specific risk was limited. Fourth, our findings apply to postmenopausal women only.

## Conclusions

In this large, prospective cohort study, the risks of ovarian cancer, particularly serous ovarian cancer, were significantly higher in the high radon zone. Approximately 25% of the US population live in homes with radon levels of more than 4.0 pCi/L.^43^ Because serous ovarian cancer is highly fatal, and because radon levels in homes can be reduced, our findings could have large implications for ovarian cancer prevention. Mechanistic hypotheses about radon and ovarian cancer are testable in laboratory animals via existing radon chambers.^44^

## Notes

Supplement 1. **eTable 1.** Missing Participants Characteristics at Baseline (Women’s Health Initiative, 1993-1998) **eTable 2.** Characteristics of WHI Participants at the Baseline Visit, by USGS Radon Zone **eTable 3.** Ovarian Tumor Subtypes Based on Histology **eTable 4.** Model Results for All Covariates From Full Ovarian Incidence Model

Supplement 2.
Data Sharing Statement
