# Supplementary material for: Residential Radon Levels and Ovarian Cancer Among Postmenopausal Women
Source: JAMA Netw Open. 2026 Apr 10;9(4):e268641. doi: 10.1001/jamanetworkopen.2026.8641 (PMC13069456; doi:10.1001/jamanetworkopen.2026.8641)
Supplement: Supplement 1. — eTable 1. Missing Participants Characteristics at Baseline (Women’s Health Initiative, 1993-1998) eTable 2. Characteristics of WHI Participants at the Baseline Visit, by USGS Radon Zone eTable 3. Ovarian Tumor Subtypes Based on Histology eTable 4. Model Results for All Covariates From Full Ovarian Incidence Model [file jamanetwopen-e268641-s001.pdf]

Supplementary Online Content

Williamson MR, Whitsel EA, Smith RL, et al. Residential radon levels and ovarian cancer among postmenopausal women. *JAMA Netw Open.* 2026;9(4):e268641. doi:10.1001/jamanetworkopen.2026.8641

eTable 1. Missing Participants Characteristics at Baseline (Women's Health Initiative, 1993-1998)

eTable 2. Characteristics of WHI Participants at the Baseline Visit, by USGS Radon Zone

eTable 3. Ovarian Tumor Subtypes Based on Histology

eTable 4. Model Results for All Covariates From Full Ovarian Incidence Model

This supplementary material has been provided by the authors to give readers additional information about their work.

© 2026 Williamson MR et al. *JAMA Network Open.*

eTable 1. Missing Participants Characteristics at Baseline (Women's Health Initiative, 1993-1998)

Characteristic	Variable	n (%)
Exposure	USGS Radon Zone	5 (0.0%)
	EPA Radon Zone	5 (0.0%)
	USGS Radon Index	5 (0.0%)
	LBL Geometric Mean	3,490 (2.2%)
Design	Clinical Trial Participation	0 (0.0%)
	Hormone Replacement Trial Arm	0 (0.0%)
Sociodemographic	Age	0 (0.0%)
	Race	0 (0.0%)
	Ethnicity	0 (0.0%)
	US Census Region	0 (0.0%)
	Education	1180 (0.7%)
	Occupation	9,934 (6.3%)
	Neighborhood SES	477 (0.3%)
Behavioral/Clinical/Reproductive	Ever Smoker	1,883 (1.2%)
	BMI	1,388 (0.9%)
	Contraceptive Use	3 (0.0%)
	Parity	805 (0.5%)
	Menarche	414 (0.3%)
	Age at Menopause	9071 (5.1%)
	Breastfeeding Months	2019 (1.3%)
	Oophorectomy	602 (0.4%)
	Hysterectomy	86 (0.1%)

© 2026 Williamson MR et al. *JAMA Network Open.*

eTable 2. Characteristics of WHI Participants at the Baseline Visit, by USGS Radon Zone

**Characteristic**	**USGS Zone**		
**Design**	**Low Radon Zone**	**Medium Radon Zone**	**High Radon Zone**
Clinical Trial Participation (yes vs. no)	21,951 (42.9%)	20,378 (42.3%)	12,084 (42.8%)
Hormone Replacement Trial Arm			
Not randomized	42,275 (42.9%)	39,796 (82.7%)	22,648 (80.2%)
Hysterectomy: Estrogen treatment	1,323 (2.6%)	1,241 (2.6%)	708 (2.5%)
Hysterectomy: Placebo	1,279 (2.5%)	1,237 (2.6%)	707 (2.5%)
Uterus: Estrogen + Progestin treatment	3,328 (6.5%)	2,973 (6.2%)	2,158 (7.6%)
Uterus: Placebo	3,072 (6.0%)	2,898 (6.0%)	2,004 (7.1%)
Sociodemographic			
Age (years)	63.3 (7.4)	63.0 (7.2)	63.0 (7.0)
Race: White vs. Non-White	43,055 (84.1%)	39,385 (81.8%)	26,680 (94.5%)
Ethnicity: Hispanic vs. Non-Hispanic	4,082 (8.0%)	1,467 (3.0%)	221 (0.8%)
US Census Region			
Northeast	10,498 (20.5%)	10,090 (21.0%)	9,615 (34.1%)
South	17,625 (34.4%)	10,768 (22.4%)	3,772 (13.4%)
Midwest	205 (0.4%)	13,594 (28.2%)	14,701 (52.1%)
West	22,848 (44.6%)	13,693 (28.4%)	137 (0.5%)
Education < College	10,867 (21.4%)	10,228 (21.4%)	6,895 (24.6%)
Occupation = Homemaker	4,473 (9.4%)	3,932 (8.5%)	2,566 (10.0%)
Neighborhood SES (z score)	-0.2 (5.6)	0.1 (5.6)	0.4 (4.8)
Behavioral/Clinical/Reproductive			
Ever Smoker	24,640 (48.8%)	23,980 (50.4%)	13,356 (47.7%)
BMI (kg/m2)	27.6 (5.8)	28.1 (6.1)	28.0 (5.7)
Contraceptive Use (ever vs. never)	20,406 (39.9%)	21,168 (44.0%)	12,238 (43.4%)
Parity			
None	5,827 (11.5%)	5,923 (12.4%)	2,854 (10.1%)
1-2	17,744 (34.1%)	16,357 (34.1%)	8,320 (29.6%)
3+	27,317 (53.7%)	25,668 (53.5%)	16,973 (60.3%)
Menarche (≤12 vs. 13+)	24,144 (47.3%)	23,083 (48.0%)	13,241 (47.0%)
Age at Menopause (years)	49.0 (5.9)	49.2 (5.8)	49.6 (5.4)
Breastfeeding Months			
None	24,477 (48.5%)	22,867 (48.0%)	13,192 (47.2%)
1-6	13,240 (26.2%)	12,214 (25.7%)	7,125 (25.5%)
7+	12,801 (25.3%)	12,531 (26.3%)	7,661 (27.4%)
Partial Oophorectomy (one/unsure vs. none)	6,340 (12.4%)	6,075 (12.6%)	3,212 (11.4%)
Hysterectomy (yes vs. no)	42,275 (42.9%)	39,796 (82.7%)	22,648 (80.2%)

© 2026 Williamson MR et al. *JAMA Network Open.*

eTable 3. Ovarian Tumor Subtypes Based on Histology

Subtype	Count (%)	Collapsed Subtype	Count (%)
Serous	785/1,657 (47%)	Serous	785/1,657 (47%)
Endometroid	93/1,657 (6%)	Non-serous	213/1,657 (13%)
Mucinous	71/1,657 (4%)		
Clear Cell	49/1,657 (3%)		
Generic	307/1,657 (19%)	Unclassified	659/1,657 (40%)
Other	97/1,657 (6%)		
Unknown	255/1,657 (15%)		
**Total**	1,657 (100%)		1,657 (100%)

© 2026 Williamson MR et al. *JAMA Network Open.*

eTable 4. Model Results for All Covariates From Full Ovarian Incidence Model

	Metrics		
**Variable**	**Hazard Ratio**	**Lower 95% CI**	**Upper 95% CI**
USGS Zone			
1 vs. 3	1.3063	1.1110	1.5360
2 vs. 3	1.1344	1.0000	1.2868
Clinical Trial Participation (yes vs. no)	0.9501	0.8389	1.0760
Hormone Replacement Trial Arm			
Hysterectomy: Estrogen treatment vs. Not randomized	0.8520	0.5821	1.2469
Hysterectomy: Placebo vs. Not randomized	0.4494	0.2665	0.7579
Uterus: Estrogen + Progestin treatment vs. Not randomized	0.9835	0.7746	1.2487
Uterus: Placebo vs. Not randomized	0.8683	0.6744	1.1178
Age (years)	1.0144	1.0059	1.0229
Race: White vs. Non-White	1.2221	1.0144	1.4723
Ethnicity: Hispanic vs. Non-Hispanic	0.7722	0.5515	1.0812
US Census Region			
South vs. Northeast	1.2146	1.0388	1.4202
Midwest vs. Northeast	0.9983	0.8501	1.1723
West vs. Northeast	1.1833	1.0113	1.3845
Education (< College vs. >= College)	1.0801	0.9425	1.2377
Occupation (Homemaker vs. Other)	0.9438	0.7843	1.1358
Neighborhood SES (z score)	1.0060	0.9953	1.0169
Smoking (ever Smoker vs. non-smoker)	1.1328	1.0204	1.2576
BMI (kg/m2)	1.0003	0.9908	1.0099
Contraceptive Use (ever vs. never)	0.8583	0.7647	0.9633
Parity			
1-2 vs. None	0.7750	0.6485	0.9262
3+ vs. None	0.7610	0.6375	0.9084
Menarche (≤12 vs. 13+)	0.9915	0.8931	1.1007
Age at Menopause (years)	1.0071	0.9973	1.0169
Breastfeeding Months			
1-6 vs. None	0.9799	0.8550	1.1229
7+ vs. None	0.9928	0.8655	1.1388
Partial Oophorectomy (one/unsure vs. none)	0.6266	0.5158	0.7612
Hysterectomy (yes vs. no)	0.5944	0.5232	0.6753

© 2026 Williamson MR et al. *JAMA Network Open.*
