# Supplementary material for: Residential Radon Levels and Ovarian Cancer Among Postmenopausal Women
Source: JAMA Netw Open. 2026 Apr 10;9(4):e268641. doi: 10.1001/jamanetworkopen.2026.8641 (PMC13069456; doi:10.1001/jamanetworkopen.2026.8641)
Supplement: Supplement 2. — Data Sharing Statement [file jamanetwopen-e268641-s002.pdf]

Data Sharing Statement

Williamson. Residential Radon Levels and Ovarian Cancer Among Postmenopausal Women. *JAMA Netw Open*. Published April 10, 2026. doi:10.1001/jamanetworkopen.2026.8641

Data
Data available: No

Additional Information
Explanation for why data not available: Researchers interested in working with WHI data should reach out to the WHI.
